# Up-to-date and projected estimates of survival for people with cystic fibrosis using baseline characteristics: A longitudinal study using UK patient registry data^☆^

**DOI:** 10.1016/j.jcf.2017.11.019

**Published:** 2018-03

**Authors:** Ruth H. Keogh, Rhonda Szczesniak, David Taylor-Robinson, Diana Bilton

**Affiliations:** a Department of Medical Statistics, London School of Hygiene and Tropical Medicine, Keppel Street, London WC1E 7HT, United Kingdom; b Division of Biostatistics and Epidemiology, Cincinnati Children's Hospital Medical Center, MLC 5041, 3333 Burnet Ave, Cincinnati 45229, OH, United States; c Department of Public Health and Policy, Farr Institute@HERC, University of Liverpool, Liverpool L69 3GB, United Kingdom; d Faculty of Medicine, National Heart and Lung Institute, Imperial College London, Guy Scadding Building, Cale Street, London SW3 6LY, United Kingdom

**Keywords:** CFTR genotype, Cystic fibrosis, Flexible parametric survival model, Longitudinal study, Patient registry, Survival

## Abstract

**Background:**

Cystic fibrosis (CF) is the most common inherited disease in Caucasians, affecting around 10,000 individuals in the UK today. Prognosis has improved considerably over recent decades with ongoing improvements in treatment and care. Providing up-to-date survival predictions is important for patients, clinicians and health services planning.

**Methods:**

Flexible parametric survival modelling of UK CF Registry data from 2011 to 2015, capturing 602 deaths in 10,428 individuals. Survival curves were estimated from birth; conditional on reaching older ages; and projected under different assumptions concerning future mortality trends, using baseline characteristics of sex, CFTR genotype (zero, one, two copies of F508del) and age at diagnosis.

**Findings:**

Male sex was associated with better survival, as was older age at diagnosis, but only in F508del non-homozygotes. Survival did not differ by genotype among individuals diagnosed at birth. Median survival ages at birth in F508del homozygotes were 46 years (males) and 41 years (females), and similar in non-homozygotes diagnosed at birth. F508del heterozygotes diagnosed aged 5 had median survival ages of 57 (males) and 51 (females). Conditional on survival to 30, median survival age rises to 52 (males) and 49 (females) in homozygotes. Mortality rates decreased annually by 2% during 2006–2015. Future improvements at this rate suggest median survival ages for F508del homozygous babies of 65 (males) and 56 (females).

**Interpretation:**

Over half of babies born today, and of individuals aged 30 and above today, can expect to survive into at least their fifth decade.

**Research in context:**

**Evidence before this study**

We searched PubMed with terms “(cystic fibrosis survival) and (projection OR model OR registry OR United Kingdom OR UK)” to identify relevant studies on survival estimates for individuals with cystic fibrosis (CF). We also considered the most recent annual report from the UK Cystic Fibrosis Registry (Cystic Fibrosis Trust, 2016), a review by Buzzetti and colleagues (2009), the chapter on Epidemiology of Cystic Fibrosis by MacNeill (2016), the study of MacKenzie and colleagues (2014), and references therein. There have been many studies of factors associated with survival in CF; most have focused on identifying risk factors, and only a few have presented estimated survival curves, which are the focus of this work. The most recent study of survival in the UK is by Dodge and colleagues (2007), who used data obtained from CF clinics and the national death register, and gave an estimate of survival for babies born in 2003. We found no previous studies that have obtained detailed information on survival using UK Cystic Fibrosis Registry data. Jackson and colleagues obtained survival estimates for the US and Ireland using registry data (Jackson et al., 2011). MacKenzie and colleagues used US Cystic Fibrosis Foundation Patient Registry data from 2000 to 2010 to project survival for children born and diagnosed with CF in 2010, accounting for sex, genotype and age at diagnosis (MacKenzie et al., 2014). Previous studies on estimated survival in CF have become out of date or have not accounted for the full range of patient characteristics available at birth. Few have presented conditional survival estimates (Dodge et al., 2007).

**Added value of this study**

This is the first study to yield detailed survival statistics using the UK Cystic Fibrosis Registry, which is one of the largest national CF registries outside of the US and has almost complete coverage of the UK CF population. The primary goal was to leverage the long-term follow-up of the nearly complete UK CF population available in the Registry for the purposes of producing accurate, precise predictions in the modern era of CF care. Estimates are presented from birth and conditional on survival to older ages. These are the first conditional estimates in CF to also account for genotype, sex and age at diagnosis, which were each included in the modelling using a flexible approach. Projections are also provided under different scenarios based on downward trends in mortality rates. Our use of flexible parametric survival models is novel in this field, and our approach could be used to provide modern survival statistics for other chronic diseases and disorders.

**Implications of all the available evidence**

Our estimates of future survival in CF under a range of different scenarios are based on data on nearly all individuals living with the disease in the UK in recent times, reflective of a modern era of care, and are most appropriate for the families of babies being born in the present day with CF. Conditional estimates inform patients who have already reached an older age, and their clinicians. Over half of babies born today, and of individuals aged 30 years and above alive today, can expect to survive into their fifth decade. Insights based on our survival projections can be used to inform future needs in CF health care provision.

## 1 Introduction

Cystic fibrosis (CF) is the most common inherited disease in Caucasian populations, affecting around 10,000 individuals in the UK today [1]. Most patients with CF die prematurely from respiratory failure and require support from healthcare services from diagnosis onwards [2]. Survival of people with CF has improved considerably over recent decades due to improvements in treatment and care and the estimated median survival age is one of the headline results reported in national registry reports. Information on predicted survival based on up-to-date data is important for people with CF, their families and the health professionals who care for them [3], [4], [5].

Survival estimates for people with CF are typically limited to median survival age at birth, stratified by sex, for instance, 48 years for males and 43 years for females in the UK [1]. However, these estimates are of limited use to patients who have already survived to a given age, and they do not take into account the full range of patient characteristics available at birth. Furthermore, current survival estimates in CF do not account for ongoing improvements in treatment and care. Mortality rates continue to decrease over time and this is anticipated to continue in the future, not least due to new targeted therapies [6], [7].

We therefore use novel survival analysis approaches to make projections of survival for babies born with CF in the present day, and for people who have survived to particular ages. We do this while taking into account baseline patient characteristics, and decreasing mortality trends over time, making use of the long term follow-up in the UK Cystic Fibrosis Registry, which is one of the largest national CF registries outside of the US and has almost complete coverage of the UK CF population [8]. Our aim is to provide more individually relevant information on survival for people with CF in the UK and projections that are relevant for planning future health care resource needs. Summary survival statistics are accompanied by clear interpretations and estimates of their uncertainty.

## 2 Methods

### 2.1 Study design and data source

This cohort study used longitudinal data from the UK CF Registry collected between 2006 and 2015. The UK CF Registry is a secure centralised database, sponsored and managed by the Cystic Fibrosis Trust, which records data on the demographics, health and treatment of almost all people with CF in the UK [8]. The analysis progressed in two parts. For the main analysis we used data on all individuals followed in the UK CF Registry during the 5-year period from 1st January 2011 to 31st December 2015 to estimate survival based on the latest age-specific mortality rates. Covariates in this analysis were sex, CFTR genotype (coded as the number of F508del alleles: two (homozygous), one (heterozygous), zero copies), and age at diagnosis. For our second analysis, in order take into account secular trends in survival, we used 10 years of longitudinal follow-up data, from 1st January 2006 to 31st December 2015, in order to obtain a more precise estimate of changes in hazards with calendar time.

Individuals who did not die during the analysis period were right-censored as follows. For those observed in the registry in 2015, we used their age at 31st December 2015 as the age of censoring; for those who were lost to follow-up, we used their age at the 31st December on their last year of providing data to the Registry plus two years. Individuals who were not seen for a period in the middle of the study period and then reappeared were considered to be observed for the full intervening time in the analysis. Ages were left-truncated at the later of: age at 1st January 2011 (main analysis) or age at 1st January 2006 (second analysis), age of diagnosis, or age at first joining the Registry. Ages at death or censoring and at left-truncation time were calculated using differences between relevant dates. Dates of birth and death were available in “month-year” format and the day was taken as the 15th of the month.

### 2.2 Statistical analysis

First we summarized the study population by sex, F508del status and age at diagnosis. We also summarized the survival data by number of deaths, births, new diagnoses and losses-to-follow-up, and person years at risk.

For all survival analyses, age was used as the time scale; [9] we used the period approach to yield up-to-date projections of long-term survival [10], [11]. We used flexible parametric survival models adjusting for baseline covariates [12]. Age at diagnosis was modelled using a restricted cubic spline. Flexible parametric survival models have the advantage of providing smooth flexible survivor curves. They use restricted cubic splines to model the log baseline cumulative hazard to achieve a good fit to the data, which may not be achievable using the standard set of parametric survival models (e.g. Weibull, log-logistic). The methodology and model building strategy are described in the Supplementary Materials (S1, S2). Our final model was used to obtain survival curves by sex, F508del status and age at diagnosis. Conditional survivor curves were also obtained (see Supplementary Materials S2), conditional on survival to ages 20, 30, 40, 50. Model fit was assessed by comparisons with Kaplan-Meier curves (Supplementary Materials S5) [13].

Estimates from the main analysis are based on the assumption that current mortality rates will continue to apply in the future, which does not account for potential future decreases in mortality rates due to improved treatment and care. Our final model was extended to enable projection of future survivor curves under different assumptions about how mortality rates may improve (Supplementary Materials S3). We provide results based on two projections: (1) improvements in mortality rates will continue at the same rate as estimated over the ten years 2006–2015 (2% reduction in mortality rates per year); (2) improvements in mortality rates will continue at half that rate (1%). The annual change in age-specific mortality rates was estimated by fitting our final selected model with an additional linear term in calendar year. For this we used data from 2006 to 2015, with the aim of obtaining a more precise estimate of changes in hazards with calendar time. The estimated hazard ratio for each calendar year was used to modify the flexible parametric survivor curves under the above two scenarios for future mortality rates. These are the same scenarios as were considered by MacKenzie and colleagues [14].

## 3 Results

### 3.1 Population characteristics

10,428 individuals (95.3%) had data on all covariates and were included in the analysis. We excluded 414 individuals (3.8%) with missing F508del genotype status and 129 individuals (1.2%) with missing age at diagnosis. The median age among those alive at the start of 2011 was 18.1 (IQR: (9.3–27.6), range: (0, 81.4)). During the 5-year period 2011–2015, there were 602 deaths, 1215 births, and 1569 new diagnoses (including newborn diagnoses). Only 142 individuals (1.4%) were defined as being lost to follow-up during the 5-year period.

Table 1 shows the baseline characteristics of the study sample used for the main analysis (2011–2015). There were slightly fewer females than males (47.1% versus 52.9%). Half of individuals (51.0%) were F508del homozygous and 39.4% were F508del heterozygous. The majority (67.2%) were diagnosed under age one. Median age at diagnosis was 0.2 years, with range 0 to 79 years. See Supplementary Table 1 for summary statistics for 2006–2015.

**Table 1:** Descriptive statistics for included individuals observed in the UK Cystic Fibrosis Registry between 1st January 2011 and 31st December 2015 (N = 10,428).

	N	%	No. deaths	Person years of follow-up
*Sex*				
Male	5519	52.9	295	24,291.9
Female	4909	47.1	307	21,481.3

*F508del status*				
Homozygous (2 copies)	5314	51.0	336	23,711.4
Heterozygous (1 copy)	4107	39.4	214	17,793.5
0 copies	1007	9.7	52	4268.3

*Age at diagnosis*				
Median, IQR	0.2	(0.06, 2.03)		
< 1	7006	67.2	373	30,529.6
1–4	1792	17.2	129	8363.7
5–9	488	4.7	29	2205.4
10–24	570	5.5	21	2511.0
25 +	572	5.5	50	2163.6

### 3.2 Models for survival

**Fig. 1:**
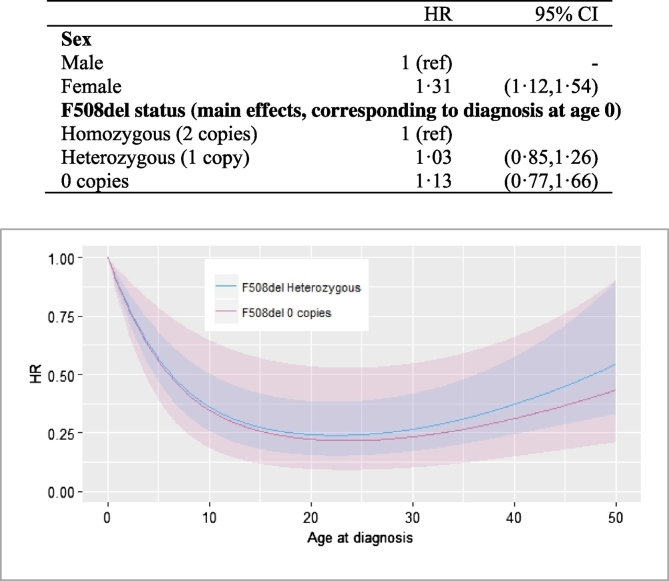
Final model results: Estimated hazard ratios (HR) and corresponding 95% confidence intervals (CI). The hazard ratios for age at diagnosis are shown graphically, with the shaded area representing the 95% CIs.

The final selected flexible parametric survival model included sex, F508del status and interactions between age at diagnosis and being F508del heterogeneous or having no copies of F508del. Age at diagnosis was not associated with survival in the F508del homozygous group after accounting for sex. The complete specification of the model is given in the Supplementary Materials (S4). Associations between covariates and survival are summarized in Fig. 1. Females had a 31% increased hazard (HR 1.31, 95% CI (1.12, 1.54)) independently of genotype and age at diagnosis. For those diagnosed at age 0 the hazard did not differ between the three genotype groups (F508del heterozygous: HR 1.03, 95% CI (0.85, 1.26), F508del zero copies: HR 1.13 (0.77, 1.66)), but we decided to retain those terms to illustrate this point clinically and because their inclusion did not result in substantial widening of survival curve confidence intervals. In individuals with zero or one copy of F508del, older age at diagnosis was associated with a reduced hazard up to around age 15 and then levelled off. The form of the association was remarkably similar in the two groups. The final model was found to provide an excellent fit to the data (Supplementary Fig. 4).

**Fig. 2:**
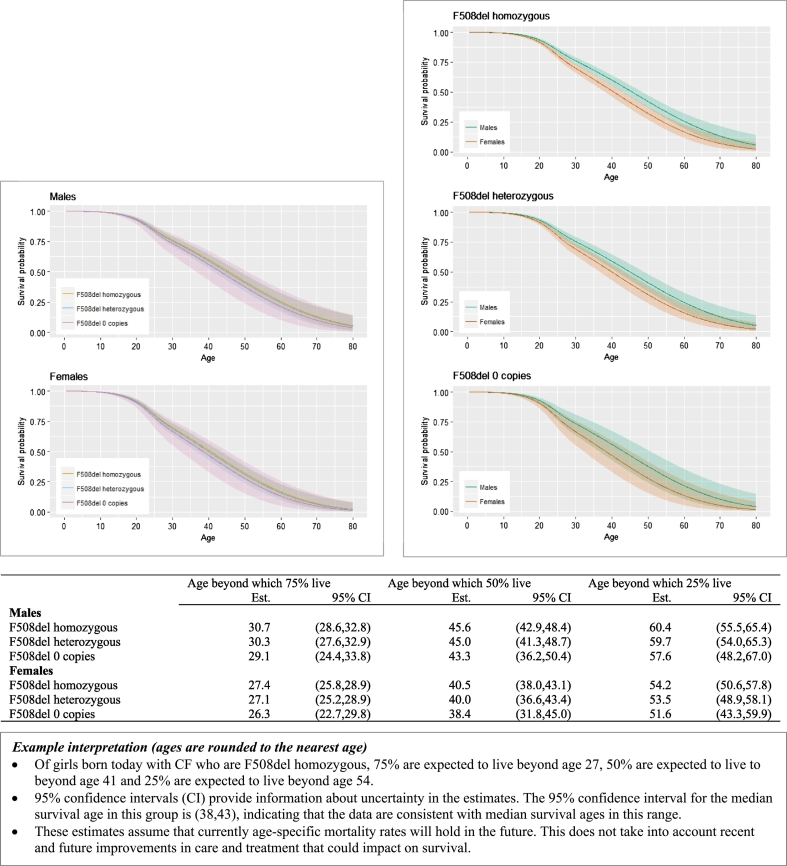
Estimated survival curves for F508del homozygous individuals (diagnosed at any age) and for individuals with 0 or 1 copy of F508del diagnosed at age 0. Left panel: by F508del status within males and females. Right panel: by sex within F508del group. The shaded areas represent the 95% confidence intervals. The table shows the estimated ages (Est), and 95% confidence intervals (CI), beyond which 75%, 50% and 25% of individuals survive in groups defined by sex and F508del status.

Results that are ages are rounded to the nearest whole age for presentation in the text. Fig. 2 shows estimated survivor curves and summary statistics, focusing on F508del homozygous individuals (diagnosed at any age) and F508del non-homozygotes diagnosed at age zero. As we expect based on the hazard ratio results (Fig. 1), the estimated curves in the three genotype groups are very similar, while the survival curves for males are consistently higher than for females. In F508del homozygotes 75% of males are expected to live beyond age 31 (95% CI 29–33), and 75% of females beyond age 27 (95% CI 26–29). The median survival ages for males and females in the F508del homozygous groups are respectively 46 (95% CI 43–48) and 41 (95% CI 38–43), and 25% of males are expected to live beyond age 60 (95% CI 56–65) and 25% of females beyond age 54 (95% CI 51–58).

### 3.3 Conditional survival curves

**Fig. 3:**
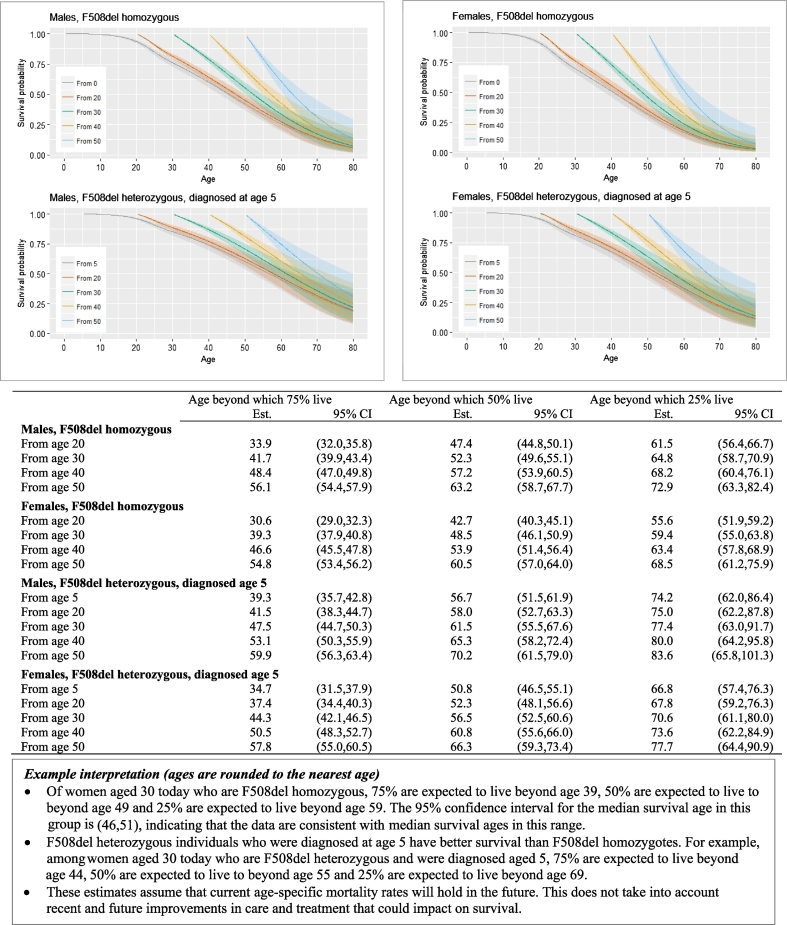
Estimated conditional survival curves for F508del homozygous individuals conditional on survival to ages 20, 30, 40, 50; and for F508del heterozygous individuals diagnosed at age 5 and conditional on survival to diagnosis and to ages 20, 30, 40, 50. Left panel: males. Right panel: females. The table shows the estimated ages (Est), and 95% confidence intervals (CI), beyond which 75%, 50% and 25% of individuals survive conditional on survival to a given age in groups defined by sex and F508del status.

Fig. 3 shows estimated conditional survivor curves for F508del homozygotes. In F508del homozygous males, the median survival age conditional on survival to age 30 is 52 (95% CI 50–55), which is an almost 7-year increase on the estimated median survival age from birth (46, 95% CI 43–48). In F508del homozygous females, the median survival age conditional on survival to age 30 is 49 (95% CI 46–51), compared to the estimated median survival age from birth of 41 (95% CI 38–43). Conditional survivor curves for individuals with zero or one copy of F508del and diagnosed at age zero are very similar to those shown for homozygous individuals and are shown in Supplementary Fig. 1.

Fig. 3 also illustrates the impact of age at diagnosis on estimated survival curves in F508del heterozygotes, via estimated survivor curves for individuals diagnosed at age 5. F508del heterozygous individuals diagnosed at age 5 have significantly improved survival compared to those diagnosed at age 0. For example, the estimated median survival age in males is 45 (95% CI 41–49) for those diagnosed at age 0 and 57 (95% CI 52–62) for those diagnosed at age 5. In females the corresponding estimates are 40 (95% CI 37–43) and 51 (95% CI 47–55). The conditional survival estimates are also considerably higher in those diagnosed at an older age, though are accompanied by increasingly wide confidence intervals due to the relatively little information on individuals with older age at diagnosis. See Supplementary Figs. 2 and 3 for results for older age at diagnosis and those with zero copies of F508del.

### 3.4 Projected survival curves

The estimated HR for a one-year increase in calendar time during 2006–2011 was 0.98 (95% CI 0.96–1.00), indicating that mortality rates reduced by 2% each year. This corresponds to a decrease of 21% (HR 0.79, 95% CI 0.64–0.97) over ten years. We focus on projections for F508del homozygous individuals, for whom age at diagnosis was not important. Projections for individuals with zero or one copy of F508del and diagnosed at age 0 are very similar under our model (Supplementary Fig. 6). However, in the light of newborn screening for CF in the UK since 2007 results for non-homozygotes would be expected to change because individuals who in the past would have been diagnosed at an older age would now be diagnosed at birth [15]. We did not consider it appropriate to present projections from birth in groups who in the past would have been diagnosed at an older age.

**Fig. 4:**
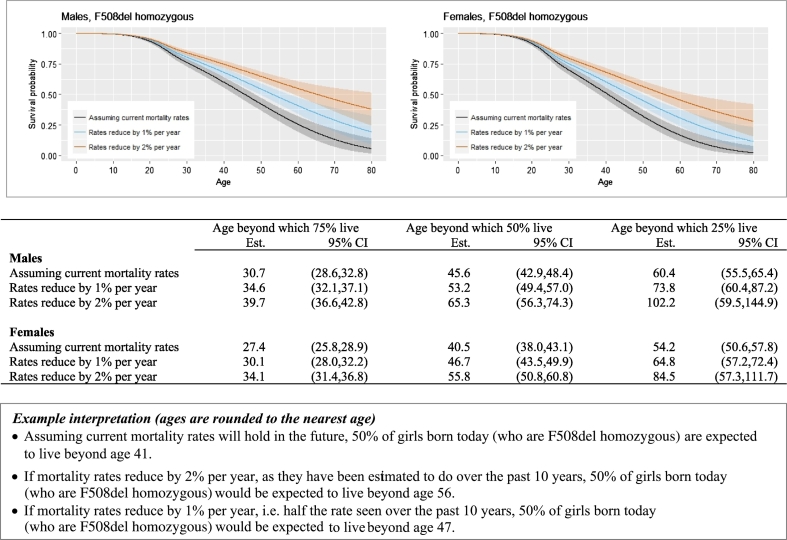
Projected survival curves for F508del homozygous individuals diagnosed at birth.

Fig. 4 shows projected survival curves. If recent trends in decreasing mortality rates continue at the same rate (2% per year) the median survival age for F508del homozygote children born today is estimated to be 65 for males (95% CI 56–74) and 56 for females (95% CI 51–61). Compared with estimates from the main analyses, which assume current mortality rates will hold in the future, these results correspond to an increase in median survival age of 20 years in males and 15 years in females. Under the conservative projection assuming hazard rates reduce by 1% per year, median survival is estimated to increase by eight years in males and six years in females. For context, these results compare with life expectancy estimates for the general UK population of 79.1 in males and 82.8 in females [16].

## 4 Discussion

We outline a novel approach to estimating survival in people with CF, using data from the UK CF Registry. We provide survival curves from birth taking into account sex, genotype and age at diagnosis, and update these estimates conditional on survival to later ages. We show that under current mortality rates and in babies born today with the most common genotype (F508del homozygous), half of males are expected to live beyond age 46 and half of females beyond age 41. If CF mortality rates continue to decrease by 2% per year this increases to 65 for males and 56 for females. These up-to-date survival statistics will be important to inform planning of health care services, given the evidence for continued improvements in survival in CF over time. They also inform patients making life and treatment decisions and clinicians making treatment choices. For individual patients who have already reached a given age, measures of current health status, for example lung function, are likely to be more important predictors of survival than baseline characteristics, and providing such estimates using the longitudinal data available in the Registry is an area of ongoing work. Our findings should be interpreted in the light of the uncertainty and assumptions involved, as highlighted in figures displaying the main results (Fig. 2, Fig. 3, Fig. 4). We refer to a complementary paper for a detailed commentary on interpretation of median survival estimates [17].

There is a large literature on survival in CF. [18], [19] Previous studies have presented survival statistics in CF based on national data though we are the first to provide detailed information on survival using the UK CF Registry. Dodge et al. [4] assessed CF survival in the UK during 1947–2003 using data from CF clinics and the national death register based on both birth cohort and current life table approaches, the latter being equivalent to the period approach employed in this study. Similar studies were performed by the same authors using earlier data [20], [21]. Elborn and colleagues used national mortality data for CF in England and Wales from 1959 to 1986 to predict survival of individuals born with CF up to 1990 [3]. MacKenzie and colleagues used US CF Foundation Patient Registry data from 2000 to 2010 to project survival for children born and diagnosed with CF in 2010 [14]. They used kernel-smoothed survivor curves obtained by Cox regression modelling, in contrast to our flexible parametric survival modelling approach, which arguably provides a more theoretically sound option for obtaining smooth estimated survival curves. Jackson and colleagues fitted parametric survival models based on a Weibull model to US Registry data from the 1980–1994 birth cohort, taking into account sex and birth cohort [5]. The parametric models developed on the US population were extrapolated to infer estimates of longer-term survival and were also applied to data from the CF Registry of Ireland. We compared our flexible models with a Weibull model and found the former to provide a better fit to the data (Supplementary Fig. 5).

In our study we estimate median survival at birth to be 46 years in males and 41 years in females for babies born with CF now who are F508del homozygous, based on the conservative assumption that mortality rates will remain at the current level. These estimates are a little lower than those reported in recent UK CF Registry Annual Report [1] (48 for males, 44 for females), which do not account for genotype or age at diagnosis. Dodge and colleagues estimated a mean age of survival of 43 in males and 37 in females for a child born with CF in 2003 [4], while Elborn and colleagues estimated median survival of age 40 for a child born with CF in 1990 [3]. These estimates did not account for genotype or age at diagnosis. Given findings presented in the UK CF annual registry reports that overall survival is improving over time, and our finding of decreasing mortality rates over time, it is logical that our up-to-date estimates for the UK are higher. Jackson and colleagues estimated median survival of 51 for males and 42 for females among individuals born during 1985–1994 in the US and who survived to their first birthday [5]. Corresponding estimates for Ireland were 51 and 39. Although based on non-UK populations, their estimates are similar to ours, but they were based on extrapolated birth cohort survivor curves, while our estimates use current mortality rates across all ages. MacKenzie and colleagues found that for a child born and diagnosed with CF in the US in 2010 the estimated median survival was 39 for males and 35 for females in the F508del homozygous group [14]. Their estimates for homozygotes are lower than ours, because they refer to 2010 whereas we used data up to the end of 2015 and because survival estimates for the US are lower than for the UK [1], [22].

Our study provides survival information for individuals living with CF who have already reached a given age, which has been extremely limited to date. Conditional estimates of survival are most relevant for patients and clinicians. Given survival to age 30 in F508del homozygotes, we found median survival age of 53 for males and 49 for females, which are increases of seven and eight years respectively compared to survival from birth. Estimated conditional median survival ages were found to be considerably higher for non-F508del homozygotes diagnosed at an older age. Dodge and colleagues presented conditional life expectancies (a mean rather than a median) for a series of ages up to age 30 for males and females separately [4]. The expectation of life in males and females who had reached age 30 in 2003 were 52 and 49 years respectively. Nick et al. presented median survival estimates for long term survivors (to age 40 plus) of CF in the US stratified by sex and age of diagnosis, finding estimated median survival ages from age 40 of 54 for those diagnosed in childhood (age ≤ 10) and 68 for those diagnosed in adulthood (age ≥ 18) [23]. These earlier estimates did not take into account genotype.

We have also produced projections of future survival in CF in the UK under two further scenarios. These estimates are most appropriate for the families of babies being born in the present day with CF, and can be used to inform future needs in CF health care provision. MacKenzie and colleagues estimated a decrease in mortality rates of 1.8% per calendar year using US Registry data from 2000 to 2010 [14], which is very similar to our estimate of 2% for the UK. Continued improvements at this rate in the future correspond to an increase in median survival age of 20 years in males and 15 years in females compared to estimates that do not take into account the secular downward trend in mortality. These increases are similar to those found by MacKenzie and colleagues, though their actual estimates were lower [14].

The baseline characteristics of sex, F508del status and age at diagnosis were incorporated into our survival estimates. Sex was associated with survival independently of the other two characteristics, and age at diagnosis was an important predictor of survival in non-F508del homozygotes but not in homozygotes. MacKenzie and colleagues found sex, F508del status and age at diagnosis to be independently associated with survival in the US setting, but did not investigate an interaction between genotype and age at diagnosis [14]. Such an interaction could be country-specific due to differing health care systems. Females have long been found to have worse survival in CF compared to males and our findings further support that phenomenon in up-to-date data [24]. More research using longitudinal data is needed to elucidate the pathways to worse outcome for females [25], [26], [27]. Older age at diagnosis has also previously been associated with better prognosis [23]. Sensitivity analyses restricting to individuals aged under 18 at diagnosis did not materially impact on the results. It is likely that the impact of age at diagnosis in the non-F508del homozygotes at least in part represents the impact of milder genotypes on the second (or both) allele [23]. We considered using genotype risk class (I, II, III, IV, V, or groupings thereof) [28] in place of F508del status. However, genotype risk class was missing in 15% of individuals (due to missing information one or both mutations, or due to an unclassified mutation) and the missingness was strongly suspected to be missing not at random. In the primary data set only six deaths were observed for individuals in classes III–V, which was insufficient to allow sensible analysis. In further investigations using the main study sample we found that in non-F508del homozygotes, 57% of those diagnosed under age five had a high risk genotype (where genotype risk class was observed), according to the genotype classification of McKone and colleagues [28], compared with only 14% of those diagnosed after age five. Further work into survival for specific sub-categories of non-F508del homozygotes would be of interest. Our genotype categorization has the advantage of being understandable for patients, based on sufficiently large numbers to obtain precise estimates, and observed for nearly all patients.

Significant strengths of the data used in this study include that there is very little loss-to-follow-up or missing data on the variables considered. Although there were some missing data on genotype and age at diagnosis, the amount of missingness was so small that it is unlikely to have had significant impact on results. Our study also has some limitations. Survival data for younger ages are based on individuals born in the era of newborn screening for CF, introduced in the UK in 2007 [15], [29], while survival data for older individuals includes a greater proportion diagnosed at an older age. Accordingly, survival data from people of older ages are necessarily based on individuals born in older birth cohorts who have not received the same care and treatments at younger ages as more recent birth cohorts. The survival curves therefore cannot fully reflect current standards of care. Reducing deaths in the childhood period is an important goal of CF care, and in our analysis 8% of deaths (N = 49) were in CF patients under the age of 18 years. Additionally, our projections do not account for emerging therapies aimed at correcting the underlying defect of CFTR [6], [7], which may have differential impact in some groups of individuals; however, the more realistic scenarios and projections reported here offer insights into potential survivorship gains. Age at diagnosis was found to be an important predictor of survival in non-homozygotes, but we could not present projected survivor curves for non-homozygotes born at an older age because we did not have data on survival from birth in those types of individuals. The conditional estimates are, however, still relevant for individuals living with CF who were born in the pre-newborn screening era. Although we found that age at diagnosis was not an important factor in survival for F508del homozygotes, the availability of newborn screening is still likely to have impacted on survival for this group via improved treatment at younger ages [30].

In summary, we have used data from the UK CF Registry, which has almost complete coverage of the national CF population, to provide up-to-date and precisely estimated survival curves that can be used by clinicians to effectively convey information on mortality to patients and families. Our study applies state of the art statistical methodology using flexible parametric survival curves, enabling incorporation of baseline characteristics and extrapolation to make future predictions, while providing an excellent fit to the data. Babies born with CF in the UK today can reasonably expect to live beyond their 50th birthday, as can individuals living with CF today who have already reached age 30 or older.

It is important that patients and care-givers are supported to interpret survival estimates, the uncertainty around those estimates, and the caveats to them. Indeed, there is much scope for further qualitative work with patients and clinicians to better understand how they make use of survival data in CF, drawing on insights from the risk communication literature [31], [32].

## Author contributions

RK conducted the analyses and drafted the manuscript. RS provided input on the statistical analysis. RS, DTR and DB contributed to data interpretation, data presentation, and writing of the manuscript. All authors approved the final version of the manuscript.

## Declaration of interests

There are no conflicts of interest.

## Appendix A Supplementary material

Supplementary material Image 1
