# Supplementary material for: Up-to-date and projected estimates of survival for people with cystic fibrosis using baseline characteristics: A longitudinal study using UK patient registry data
Source: J Cyst Fibros. 2018 Mar;17(2):218–27. doi: 10.1016/j.jcf.2017.11.019 (PMC5885983; doi:10.1016/j.jcf.2017.11.019)
Supplement: Supplementary file 1 — Supplementary material [file mmc1.docx]

**Supplementary Materials**

**Up-to-date and projected estimates of survival for people with cystic fibrosis using baseline characteristics: A longitudinal study using UK patient registry data**

**Ruth H Keogh, Rhonda Szczesniak, David Taylor-Robinson, Diana Bilton**

**S1. Flexible parametric survival models: General specification**

Flexible parametric survival models were proposed by Royston and Parmar.^1^ Under a general form of flexible parametric survival model, the survivor function at time $t$ for an individual with vector of explanatory variables $x$, defined as $S\left( t | x \right)=\Pr\left( T>t | x \right)$ where $T$ is a random variable representing age of death, is modelled as

|  | $g\left( S\left( t \vert x \right) \right)=g\left( S_{0}(t) \right)+\beta^{T}x$ | S1 |
| --- | --- | --- |

where $g\left( \cdot\right)$ is some function, $S_{0}\left( t \right)$ is the baseline survivor function and $\beta$ is a vector of parameters associated with the explanatory variables. A number of different forms have been described for $g\left( \cdot\right)$. ^1^ In this paper we use a form for $g\left( \cdot\right)$ which implies the proportional hazards model, $h\left( t | x \right)=h_{0}\left( t \right)e^{\beta^{T}x}$, under which we have

|  | $\log\left\{ -\log S\left( t \vert x \right) \right\}=\log\left\{ -\log S_{0}\left( t \right) \right\}+\beta^{T}x.$ | S2 |
| --- | --- | --- |

This uses the result that the survivor and hazard function are linked via the formula $S\left( t | x \right)=\exp\left\{ -\int_{0}^{t} h\left( u | x \right)du \right\}$. Note that $\log\left\{ -\log S_{0}\left( t \right) \right\}=\log H_{0}(t)$, where $H_{0}(t)$ is the cumulative baseline hazard. In the parametric flexible survival formulation $\log\left\{ -\log S_{0}\left( t \right) \right\}$ is modelled using a restricted cubic spline for $\log t$, which we denote $sp(\log t;\gamma)$ where $\gamma$ are parameters to be estimated. The form of $sp(\log t;\gamma)$ under a restricted cubic spline with $m$ internal knots $k_{1},\ldots,k_{m}$ and boundary knots $k_{min}$ and $k_{max}$ is

|  | $sp\left( \log t;\gamma\right)=\gamma_{0}+\gamma_{1}\log t+\gamma_{2}v_{1}\left( \log t \right)+\cdots+\gamma_{m+1}v_{m}(\log t)$ | S3 |
| --- | --- | --- |

where

|  | $v_{j}\left( \log t \right)=\left( \log t-k_{j} \right)_{+}^{3}-\frac{k_{max}-k_{j}}{k_{max}-k_{min}}\left( \log t-k_{min} \right)_{+}^{3}-\frac{k_{j}-k_{min}}{k_{max}-k_{min}}\left( \log t-k_{max} \right)_{+}^{3}$ | S4 |
| --- | --- | --- |

where $\left( \log t-a \right)_{+}=\max(0,\log t-a)$. The Weibull distribution is a special case of the model in equation S2 using a restricted cubic spline with no internal or boundary knots.

Conditional survival curves are obtained using the result that the probability of survival beyond age $t$ conditional on survival to age $u$ is the ratio of the survival probabilities at ages $t$ and $u$ $S\left( t | x, T>u \right)= S\left( t | x \right)/S\left( u | x \right)$.

All analyses were performed using R. Flexible parametric survival models were fitted using the ‘flexsurv’ package.^2^ Confidence intervals for estimates of quantiles of survival curves (including median survival age) were obtained using a simulation-based approach with 10000 samples.^3^

**S2. Model building strategy**

The base flexible parametric survival models included main effects of sex (females vs males), F508del group (homozygous (reference category), heterozygous, other) and age at diagnosis, which was modelled using a restricted cubic spline with 3 knots at ages 1, 8 and 38, these being approximately the 10^th^, 50^th^ and 90^th^ percentiles of the distribution of unique ages at diagnosis. The log cumulative baseline hazard was modelled using a restricted cubic spline with 3 internal knots at the 25^th^, 50^th^ and 75^th^ percentiles of the distribution of ages at death, and boundary knots at the extremes. Building on the baseline model, pairwise interactions were investigated and interactions significant with likelihood ratio test (LRT) p-value <0.05 were included in the model. The base model assumes proportionality of hazards. This assumption was investigated by assessing evidence for between each variable and the baseline cumulative hazard time terms. We investigated whether model fit could be improved by increasing the number of knots used in the spline to model the cumulative bassline hazard. The base model included 3 internal knots and we investigated increasing this to 4 knots (at 20, 40, 60 80) or 5 knots (at 20, 40, 60 80). The number of knots giving the lowest AIC value was selected and the earlier procedure of model selection repeated to assess whether a change in the number of knots in the baseline changed any of the earlier decisions. The AIC (Akaike’s Information Criterion) is a model fit diagnostic with lower values indicating better fit.^4^ We also investigated changing the placement of the knots for the spline for age of diagnosis.

**S3. Projecting future survivor curves**

Trends in mortality rates over calendar time were estimated using an extension to model S2 to include an additional term for calendar year at age $t$ minus 2006, denoted $y(t)$

|  | $\log\left\{ -\log S\left( t \vert x \right) \right\}=\log\left\{ -\log S_{0}\left( t \right) \right\}+\beta^{T}x+\eta y(t)$ | S5 |
| --- | --- | --- |

Parameter $\eta$ is the log hazard ratio associated with each year increase in calendar time. The hazard ratio associated with a 10-year increase in calendar time is therefore $e^{10\eta}$. The model was fitted using a time-dependent variable for calendar year. The estimate of the parameter $\eta$ is denoted $\hat{\eta}$. To use this to make projections of future survival we used the following procedure. Based on the final model (see section S4) fitted using the 5-year data we obtained the estimated hazards on a fine grid of times from 0 to 200 using increments of 0.1. This was done separately in six subgroups defined by sex and F508del status, assuming diagnosis at age 0. The estimated hazard at age $a$ in a given subgroup is denoted $h_{a}$. Under Projection 2 the hazard age $a$ is multiplied by $e^{\hat{\eta}a}$. Using the Nelson-Aalen estimate, the projected survival probability at age t is given by

|  | $S_{P2}\left( t \right)=\exp\left( -\Sigma_{a\leq t}h_{a}e^{\hat{\eta}a} \right)$ | S6 |
| --- | --- | --- |

The projected survival probabilities were found separately by the subgroups noted above for each $t$ in the fine grid of times from 0 to 200. The probabilities were joined to form a curve for Figure 4 (main text) and Supplementary Figure 5. 95% confidence intervals were obtained using $S_{P2}\left( t \right)e^{\mp1.96\sqrt{\text{var}\left( \hat{H}(t) \right)}}$, where $\text{var}\left( \hat{H}(t) \right)$ denotes the estimated variance of the cumulative hazard obtained from the final model for a given subgroup.

**S4. Final model specification**

The final model is of the form

|  | $\log\left\{ -\log S\left( t \vert female,F508.1,F508.0,diag.age,sp.diag.age \right) \right\}=\hat{\gamma}_{0}+\hat{\gamma}_{1}\log t+\hat{\gamma}_{2}v_{1}\left( \log t \right)+\hat{\gamma}_{3}v_{2}\left( \log t \right)+\hat{\gamma}_{4}v_{3}\left( \log t \right)+\hat{\gamma}_{5}v_{4}\left( \log t \right)+\hat{\beta}_{F}female+\hat{\beta}_{F508.1}F508.1+\hat{\beta}_{F508.0}F508.0+\hat{\beta}_{F508.1:diag.1}F508.1\times diag.age+\hat{\beta}_{F508.1:diag.2}F508.1\times sp.diag.age{+\hat{\beta}}_{F508.0:diag.1}F508.0\times diag.age+\hat{\beta}_{F508.0:diag.2}F508.0\times sp.diag.age$ | S7 |
| --- | --- | --- |

where $'female'$ takes value 1 for females and 0 for males, $'diag.age'$ denotes age at diagnosis (in years), $'sp.diag.age'$ denotes the spline term for age at diagnosis (see below), $'F508.1'$ takes value 1 for F508del heterozygous individuals and 0 otherwise, and $'F508.0'$ takes value 1 for individuals with 0 copies of F508del and 0 otherwise. The model parameter estimates are given in Supplementary Table 2. The knots used in the

basis functions $v_{j}(\cdot)$ were (-1.789708, 3.061041, 3.261135, 3.456867, 3.744277, 4.434720). $'sp.diag.age'$ is obtained using the function

|  | $\frac{1}{\left( c_{3}-c_{1} \right)^{2}}\left\{ \left( x-c_{1} \right)_{+}^{3}-\frac{\left( x-c_{2} \right)_{+}^{3}\left( c_{3}-c_{1} \right)}{\left( c_{3}-c_{2} \right)}+\frac{\left( x-c_{3} \right)_{+}^{3}\left( c_{2}-c_{1} \right)}{\left( c_{3}-c_{2} \right)} \right\}$ | S8 |
| --- | --- | --- |

where $x$ denotes age at diagnosis (in years) and the knots are at $c_{1}=1, c_{2}=8, c_{3}=40$. These are approximately the 10^th^, 50^th^ and 90^th^ percentiles of unique ages at diagnosis in the non-F508del homozygotes. This knot placement was found to give a slightly improved model fit compared to those used in the base model and was appropriate due to our finding that age at diagnosis was not associated with survival in the F50del homozygotes.

**S5.** **Assessing model fit**

Assessments of model fit are shown in Supplementary Figure 4, via comparisons between the fitted survival curves from the flexible parametric model and Kaplan-Meier estimates of the survival curves within groups defined by sex, F508del status and age at diagnosis. These plots demonstrate that the model provides a very close fit to the data in F508del homozygous individuals. The fit appears to be slightly better in males than females. In F508del heterozygous individuals diagnosed at age 0 (under age 1 in the Kaplan-Meier estimates) the model fit remains good: although there is some minor lack of fit at younger ages (between ages 10 and 20) this will not have an important impact on estimates of survival probabilities at older ages, which is where the majority of deaths occur. The number of individuals with 0 copies of F508del is small, particular when reduced to those with older age at diagnosis, and the confidence intervals for the Kaplan-Meier curves are very wide. However, the estimated curves show a close fit to the Kaplan-Meier estimates in females, and only minor deviation in males. The plots demonstrate how a flexible parametric model-based approach can provide more precise estimated survivor curves compared with the non-parametric Kaplan-Meier approach.

We repeated the analysis using a Weibull form for the baseline cumulative hazard instead of the flexible form. Graphical comparisons between the Weibull model and the Kaplan-Meier estimates are shown in Supplementary Figure 5 for F508del homozygotes. The plots demonstrate that, although the Weibull model fit is quite good, the flexible parametric model provides a superior fit. The AIC^4^ from the Weibull model was 5905.39, compared with 5860.522 from the flexible parametric model, indicating that the flexible parametric model provides a better fit.

**Supplementary Table 1. Descriptive statistics for included individuals observed in the UK Cystic Fibrosis Registry between 1^st^ January 2006 and 31^st^ December 2015 (N=11096). During this 10-year period there were 1106 deaths, 2558 births, and 3344 new diagnoses (including newborn diagnoses), and 306 individuals (2.8%) were defined as being lost to follow-up.**

|  | N | % | No. deaths | Person years of follow-up |
| --- | --- | --- | --- | --- |
| **Sex** |  |  |  |  |
| Male | 5842 | 52.6 | 524 | 44240.6 |
| Female | 5254 | 47.4 | 582 | 38881.0 |
| **F508del status** |  |  |  |  |
| Homozygous (2 copies) | 5709 | 51.5 | 656 | 43979.0 |
| Heterozygous (1 copy) | 4338 | 39.1 | 376 | 31792.2 |
| 0 copies | 1049 | 9.5 | 74 | 7350.4 |
| **Age at diagnosis** |  |  |  |  |
| Median, IQR | 0.2 | (0.06,2.03) |  |  |
| <1 | 7421 | 66.9 | 699 | 54683.4 |
| 1-4 | 1951 | 17.6 | 249 | 16358.7 |
| 5-9 | 521 | 4.7 | 55 | 4083.8 |
| 10-24 | 605 | 5.5 | 34 | 4549.9 |
| 25+ | 598 | 5.4 | 69 | 3445.9 |

**Supplementary Table 2. Parameter estimates (Est) from the final model and 95% confidence intervals (CI)**

|  | **Est** | **95% CI** |
| --- | --- | --- |
| $\gamma_{0}$ | -9.48 | (-10.73,-8.23) |
| $\gamma_{1}$ | 0.28 | (-0.27,0.83) |
| $\gamma_{2}$ | -10.36 | (-14.58,-6.15) |
| $\gamma_{3}$ | 19.61 | (8.43,30.79) |
| $\gamma_{4}$ | -8.82 | (-19.32,1.67) |
| $\gamma_{5}$ | -0.72 | (-5.11,3.68) |
| $\beta_{F}$ | 0.27 | (0.11,0.43) |
| $\beta_{F508.1}$ | 0.03 | (-0.17,0.23) |
| $\beta_{F508.0}$ | 0.12 | (-0.26,0.51) |
| $\beta_{F508.1:diag.1}$ | -0.12 | (-0.15,-0.08) |
| $\beta_{F508.1:diag.2}$ | 0.28 | (0.18,0.39) |
| $\beta_{F508.0:diag.1}$ | -0.12 | (-0.19,-0.05) |
| $\beta_{F508.0:diag.2}$ | 0.28 | (0.11,0.46) |

**Supplementary Figure 1. Estimated conditional survival curves for F508del heterozygous and F508del 0 copies individuals diagnosed at age 0 conditional on survival to ages 20, 30, 40, 50. Left panel: males. Right panel: females. The table shows the estimated ages (Est), and 95% confidence intervals (CI), beyond which 75%, 50% and 25% of individuals survive conditional on survival to a given age in groups defined by sex and F508del status.**


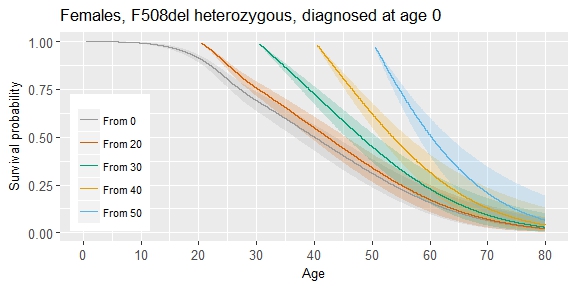


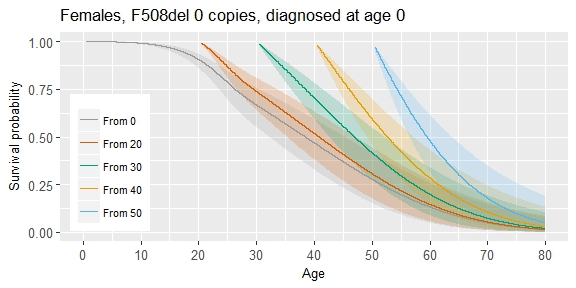


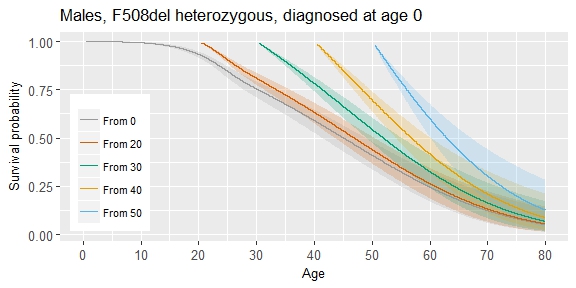


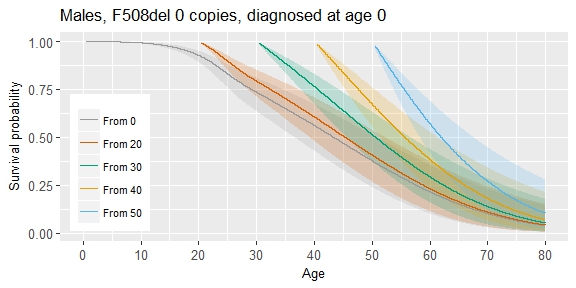


|  | Age beyond which 75% live | | Age beyond which 50% live | | Age beyond which 25% live | |
| --- | --- | --- | --- | --- | --- | --- |
|  | Est. | 95% CI | Est. | 95% CI | Est. | 95% CI |
| **Males, F508del heterozygous, diagnosed age 0** | | | | | | |
| From age 20 | 33.4 | (31.0,35.9) | 46.8 | (43.2,50.4) | 61.2 | (55.1,67.3) |
| From age 30 | 41.3 | (39.2,43.4) | 51.9 | (48.4,55.4) | 64.7 | (57.5,71.9) |
| From age 40 | 48.2 | (46.5,50.0) | 57.0 | (53.2,60.9) | 68.4 | (59.8,76.9) |
| From age 50 | 56.1 | (54.2,58.1) | 63.3 | (58.2,68.4) | 73.2 | (62.3,84.1) |
|  |  |  |  |  |  |  |
| **Females, F508del heterozygous, diagnosed age 0** | | | | | | |
| From age 20 | 30.4 | (28.3,32.5) | 42.3 | (39.0,45.6) | 55.3 | (50.5,60.1) |
| From age 30 | 39.1 | (37.3,40.9) | 48.2 | (45.2,51.2) | 59.3 | (54.1,64.6) |
| From age 40 | 46.5 | (45.1,48.0) | 53.8 | (50.8,56.8) | 63.5 | (57.1,69.9) |
|  | 54.8 | (53.2,56.4) | 60.6 | (56.7,64.6) | 68.9 | (60.6,77.2) |
|  |  |  |  |  |  |  |
| **Males, F508del 0 copies, diagnosed age 0** | | | | | | |
| From age 20 | 32.3 | (27.8,36.8) | 45.2 | (38.5,51.8) | 59.0 | (49.4,68.7) |
| From age 30 | 40.5 | (37.0,43.9) | 50.5 | (44.6,56.4) | 62.7 | (53.0,72.4) |
| From age 40 | 47.6 | (44.8,50.4) | 55.8 | (50.3,61.3) | 66.5 | (56.2,76.9) |
| From age 50 | 55.6 | (53.1,58.2) | 62.3 | (56.4,68.2) | 71.6 | (60.2,83.0) |
|  |  |  |  |  |  |  |
| **Females, F508del 0 copies, diagnosed age 0** | | | | | | |
| From age 20 | 29.5 | (25.7,33.2) | 40.8 | (34.7,46.9) | 53.3 | (45.0,61.7) |
| From age 30 | 38.4 | (35.4,41.4) | 47.0 | (41.8,52.1) | 57.6 | (49.5,65.6) |
| From age 40 | 46.0 | (43.7,48.3) | 52.8 | (48.2,57.4) | 62.0 | (53.6,70.3) |
| From age 50 | 54.4 | (52.3,56.5) | 59.8 | (55.2,64.6) | 67.6 | (58.4,76.8) |

**Supplementary Figure 2. Estimated conditional survival curves for F508del heterozygous individuals diagnosed at ages 5 and 10, conditional on survival to diagnosis and to ages 20, 30, 40, 50. Left panel: males. Right panel: females. The table shows the estimated ages (Est), and 95% confidence intervals (CI), beyond which 75%, 50% and 25% of individuals survive conditional on survival to a given age in groups defined by sex and age at diagnosis.**


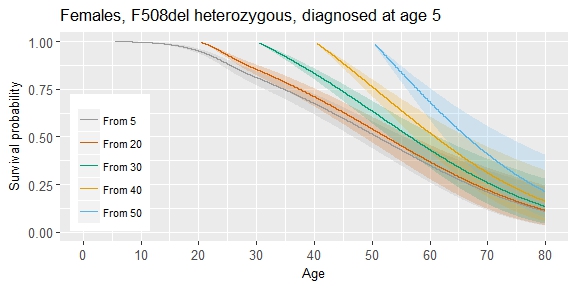


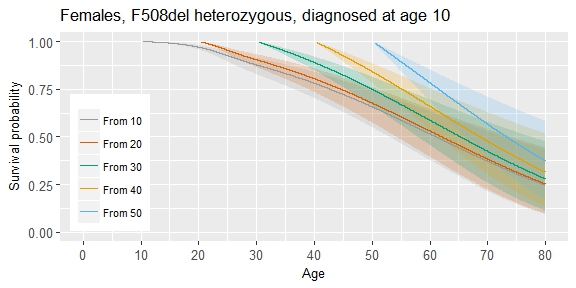


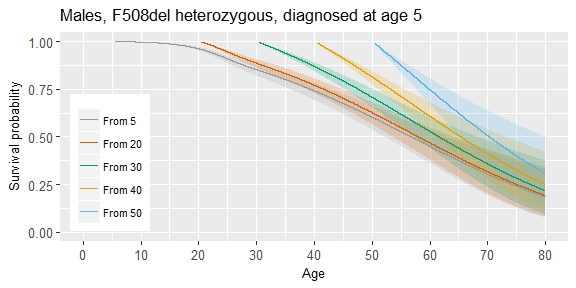


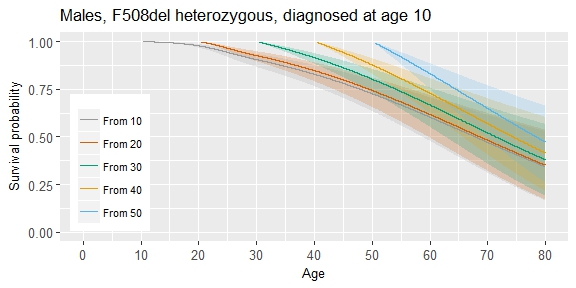


|  | Age beyond which 75% live | | Age beyond which 50% live | | Age beyond which 25% live | |
| --- | --- | --- | --- | --- | --- | --- |
|  | Est. | 95% CI | Est. | 95% CI | Est. | 95% CI |
| **Males, F508del heterozygous, diagnosed age 5** | | | | | | |
| From age 5 | 39.3 | (35.7,42.8) | 56.7 | (51.5,61.9) | 74.2 | (62.0,86.4) |
| From age 20 | 41.5 | (38.3,44.7) | 58.0 | (52.7,63.3) | 75.0 | (62.2,87.8) |
| From age 30 | 47.5 | (44.7,50.3) | 61.5 | (55.5,67.6) | 77.4 | (63.0,91.7) |
| From age 40 | 53.1 | (50.3,55.9) | 65.3 | (58.2,72.4) | 80.0 | (64.2,95.8) |
| From age 50 | 59.9 | (56.3,63.4) | 70.2 | (61.5,79.0) | 83.6 | (65.8,101.3) |
|  |  |  |  |  |  |  |
| **Females, F508del heterozygous, diagnosed age 5** | | | | | | |
| From age 5 | 33.6 | (29.6,37.5) | 49.4 | (44.2,54.6) | 65.0 | (55.0,75.1) |
| From age 20 | 36.4 | (32.9,39.9) | 51.0 | (45.9,56.0) | 66.0 | (56.0,76.1) |
| From age 30 | 43.5 | (40.7,46.4) | 55.4 | (50.1,60.6) | 68.9 | (57.4,80.5) |
| From age 40 | 49.9 | (47.3,52.4) | 59.8 | (53.7,65.8) | 72.1 | (59.2,84.9) |
| From age 50 | 57.3 | (54.3,60.2) | 65.4 | (58.2,72.7) | 76.3 | (62.3,90.2) |
|  |  |  |  |  |  |  |
| **Males, F508del heterozygous, diagnosed age 10** | | | | | | |
| From age 10 | 47.9 | (41.9,53.8) | 67.8 | (56.3,79.2) | 88.3 | (64.4,112.1) |
| From age 20 | 49.4 | (43.6,55.1) | 68.6 | (56.8,80.4) | 88.8 | (64.3,113.4) |
| From age 30 | 54.0 | (48.8,59.3) | 71.4 | (59.1,83.7) | 90.6 | (65.5,115.7) |
| From age 40 | 58.6 | (53.0,64.2) | 74.3 | (60.4,88.3) | 92.6 | (65.4,119.9) |
| From age 50 | 64.4 | (57.8,71.0) | 78.4 | (62.7,94.0) | 95.5 | (66.8,124.3) |
|  |  |  |  |  |  |  |
| **Females, F508del heterozygous, diagnosed age 10** | | | | | | |
| From age 10 | 42.7 | (37.2,48.1) | 61.0 | (52.2,69.8) | 79.6 | (60.8,98.3) |
| From age 20 | 44.5 | (39.5,49.5) | 62.0 | (53.2,70.8) | 80.2 | (61.9,98.6) |
| From age 30 | 49.9 | (45.6,54.2) | 65.2 | (56.2,74.3) | 82.3 | (63.4,101.3) |
| From age 40 | 55.1 | (50.8,59.4) | 68.6 | (58.2,79.0) | 84.7 | (63.7,105.7) |
| From age 50 | 61.5 | (56.6,66.4) | 73.2 | (61.5,84.9) | 88.0 | (65.7,110.3) |

**Supplementary Figure 3. Estimated conditional survival curves for individuals with 0 copies of F508del** **diagnosed at ages 5 and 10, conditional on survival to diagnosis and to ages 20, 30, 40, 50. Left panel: males. Right panel: females. The table shows the estimated ages (Est), and 95% confidence intervals (CI), beyond which 75%, 50% and 25% of individuals survive conditional on survival to a given age in groups defined by sex and age at diagnosis.**


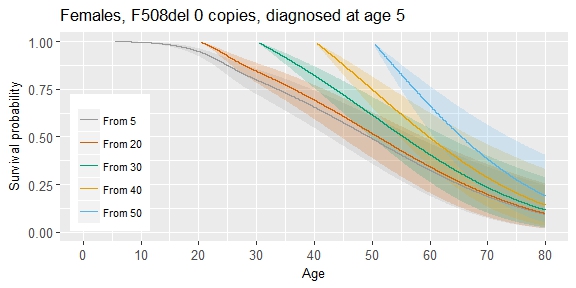


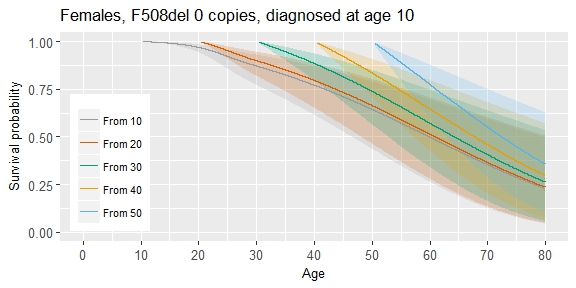


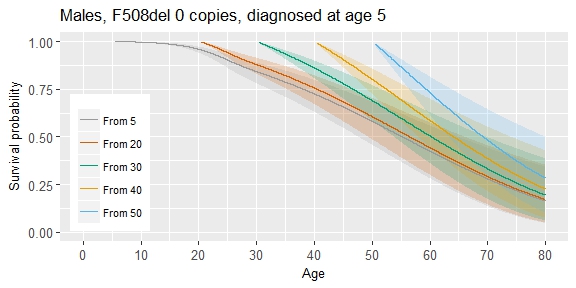


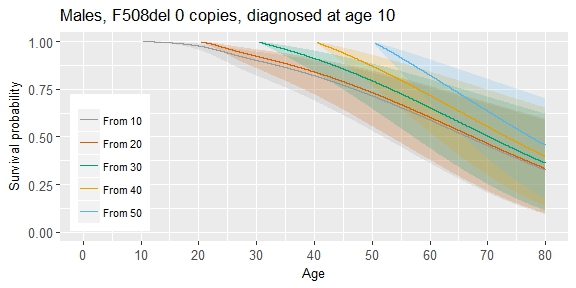


|  | Age beyond which 75% live | | Age beyond which 50% live | | Age beyond which 25% live | |
| --- | --- | --- | --- | --- | --- | --- |
|  | Est. | 95% CI | Est. | 95% CI | Est. | 95% CI |
| **Males, F508del 0 copies, diagnosed age 5** | | | | | | |
| From age 5 | 38.0 | (32.0,44.1) | 55.2 | (46.8,63.6) | 72.3 | (57.3,87.2) |
| From age 20 | 40.4 | (34.9,45.9) | 56.5 | (48.2,64.7) | 73.1 | (58.0,88.2) |
| From age 30 | 46.6 | (42.2,51.1) | 60.2 | (52.0,68.4) | 75.5 | (59.6,91.5) |
| From age 40 | 52.4 | (48.3,56.4) | 64.1 | (55.2,73.0) | 78.2 | (61.1,95.4) |
| From age 50 | 59.3 | (54.8,63.7) | 69.2 | (58.9,79.4) | 82.0 | (63.2,100.8) |
|  |  |  |  |  |  |  |
| **Females, F508del 0 copies, diagnosed age 5** | | | | | | |
| From age 5 | 33.6 | (28.2,39.0) | 49.4 | (42.1,56.7) | 65.0 | (53.6,76.5) |
| From age 20 | 36.4 | (31.4,41.4) | 51.0 | (43.8,58.1) | 66.0 | (54.2,77.9) |
| From age 30 | 43.5 | (39.7,47.4) | 55.4 | (48.6,62.1) | 68.9 | (56.4,81.5) |
| From age 40 | 49.9 | (46.6,53.2) | 59.8 | (52.8,66.8) | 72.1 | (58.4,85.7) |
| From age 50 | 57.3 | (53.9,60.6) | 65.4 | (57.7,73.2) | 76.3 | (61.9,90.7) |
|  |  |  |  |  |  |  |
| **Males, F508del 0 copies, diagnosed age 10** | | | | | | |
| From age 10 | 46.9 | (36.3,57.5) | 66.5 | (50.1,82.9) | 86.6 | (59.2,114.0) |
| From age 20 | 48.5 | (38.4,58.6) | 67.4 | (50.2,84.5) | 87.2 | (60.3,114.0) |
| From age 30 | 53.2 | (44.3,62.1) | 70.2 | (53.1,87.3) | 89.0 | (61.9,116.2) |
| From age 40 | 57.9 | (49.4,66.4) | 73.2 | (55.8,90.6) | 91.1 | (64.7,117.6) |
| From age 50 | 63.9 | (55.1,72.6) | 77.4 | (59.0,95.7) | 94.1 | (67.1,121.0) |
|  |  |  |  |  |  |  |
| **Females, F508del 0 copies, diagnosed age 10** | | | | | | |
| From age 10 | 41.8 | (32.2,51.3) | 59.8 | (45.9,73.8) | 78.1 | (55.3,100.8) |
| From age 20 | 43.7 | (34.7,52.7) | 60.9 | (46.8,74.9) | 78.8 | (55.8,101.7) |
| From age 30 | 49.2 | (41.7,56.8) | 64.2 | (50.3,78.1) | 80.9 | (57.6,104.3) |
| From age 40 | 54.5 | (47.6,61.5) | 67.7 | (53.6,81.7) | 83.4 | (59.7,107.0) |
| From age 50 | 61.0 | (54.2,67.8) | 72.4 | (57.9,86.8) | 86.7 | (62.9,110.6) |

**Supplementary Figure 4. Assessment of model fit. Comparison between the fitted survival curves estimated using the final flexible parametric survival model and the Kaplan-Meier estimates. In the plots for those with 1 or 0 copies of F508del the Kaplan-Meier curves were obtained in the subset of individuals diagnosed under age 1. In the plots for F508del heterozygotes diagnosed at age 5, the Kaplan-Meier curves were obtained in the subset of individuals diagnosed between ages 3 and 7. The number of individuals were too small to make similar plots for those with 0 copies of F508del.**


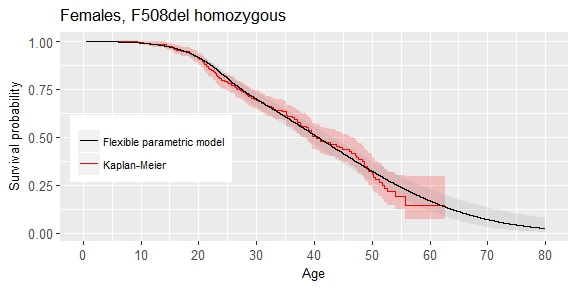


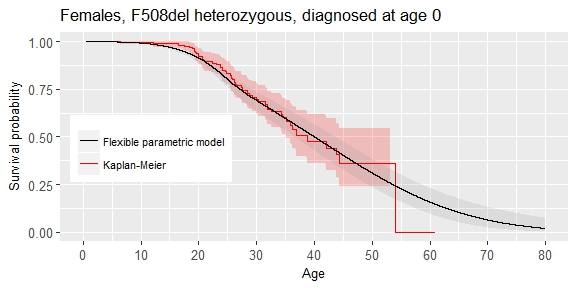


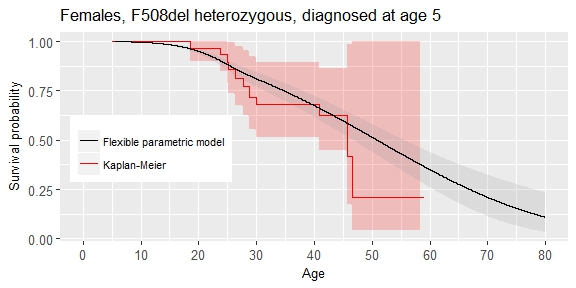


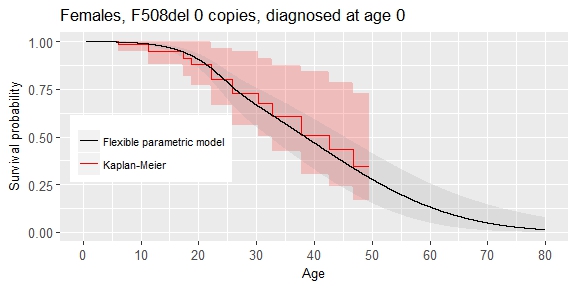


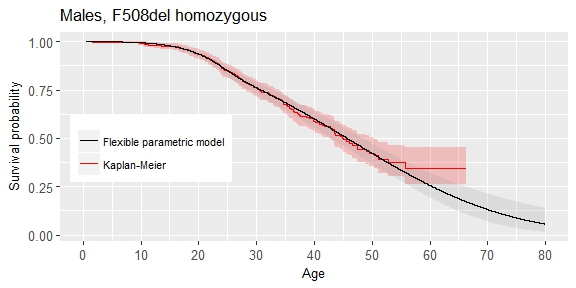


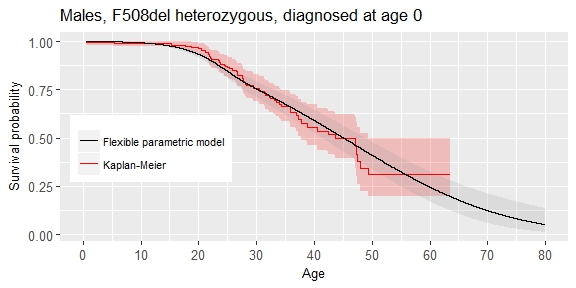


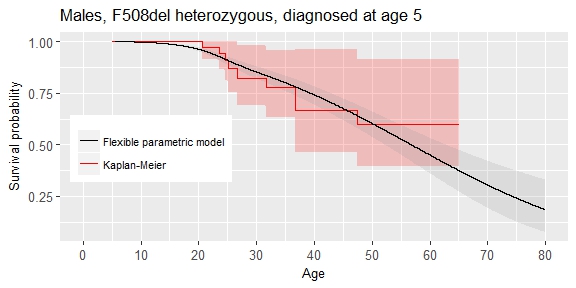


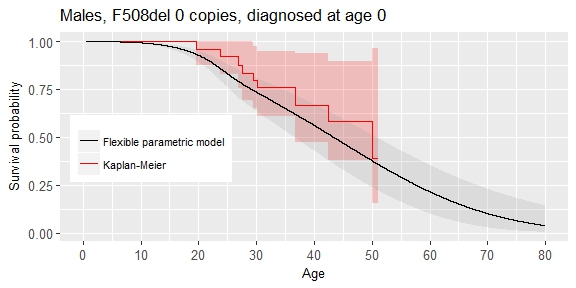


**Supplementary Figure 5. Assessment of model fit. Comparison between the fitted survival curves estimated using a Weibull form for the baseline hazard and the Kaplan-Meier estimates, for F508del homozygotes (corresponding to the first row of plots in Supplementary Figure 4).**


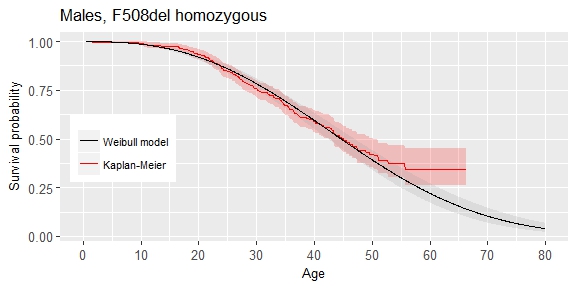


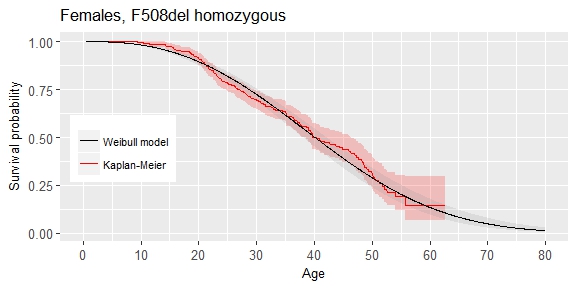


**Supplementary Figure 6. Projected survival curves for F508del heterozygous and F508del 0 copies individuals diagnosed at birth.**


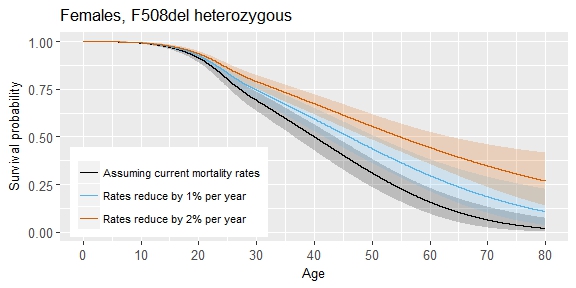


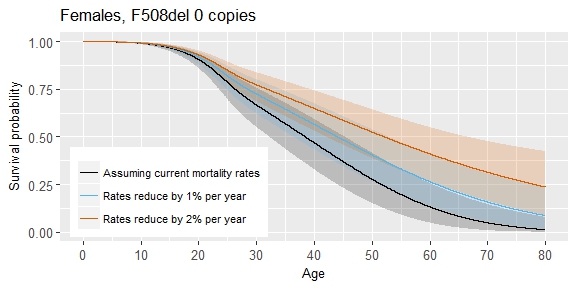


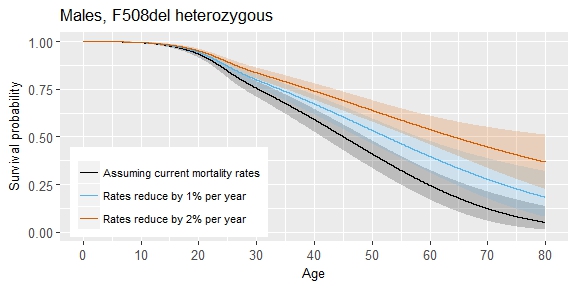


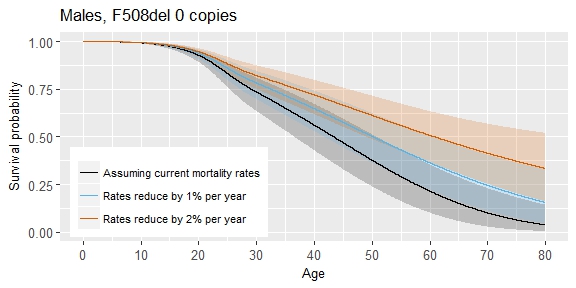


|  | Age beyond which 75% live | | Age beyond which 50% live | | Age beyond which 25% live | |
| --- | --- | --- | --- | --- | --- | --- |
|  | Est. | 95% CI | Est. | 95% CI | Est. | 95% CI |
| **Males, F508del heterozygous, diagnosed age 0** | | | | | | |
| Assuming current mortality rates | 30.3 | (27.6,32.9) | 45.0 | (41.3,48.7) | 59.7 | (54.0,65.3) |
| Rates reduce by 1% per year | 34.0 | (30.7,37.3) | 52.4 | (47.3,57.5) | 72.7 | (59.6,85.8) |
| Rates reduce by 2% per year | 39.0 | (34.8,43.2) | 64.1 | (54.2,74.0) | 99.8 | (57.2,142.4) |
|  |  |  |  |  |  |  |
| **Females, F508del heterozygous, diagnosed age 0** | | | | | | |
| Assuming current mortality rates | 27.1 | (25.2,28.9) | 40.0 | (36.6,43.4) | 53.5 | (48.9,58.1) |
| Rates reduce by 1% per year | 29.7 | (27.1,32.3) | 46.0 | (41.7,50.3) | 63.9 | (55.7,72.1) |
| Rates reduce by 2% per year | 33.5 | (30.1,36.9) | 54.8 | (48.3,61.3) | 82.8 | (56.9,108.7) |
|  |  |  |  |  |  |  |
| **Males, F508del 0 copies, diagnosed age 0** | | | | | | |
| Assuming current mortality rates | 29.1 | (24.4,33.8) | 43.3 | (36.2,50.4) | 57.6 | (48.2,67.0) |
| Rates reduce by 1% per year | 32.5 | (26.2,38.8) | 50.2 | (40.6,59.8) | 69.6 | (51.6,87.6) |
| Rates reduce by 2% per year | 37.1 | (29.0,45.2) | 60.8 | (43.8,77.8) | 93.6 | (48.6,138.6) |
|  |  |  |  |  |  |  |
| **Females, F508del 0 copies, diagnosed age 0** | | | | | | |
| Assuming current mortality rates | 26.3 | (22.7,29.8) | 38.4 | (31.8,45.0) | 51.6 | (43.3,59.9) |
| Rates reduce by 1% per year | 28.5 | (23.6,33.4) | 44.0 | (35.6,52.4) | 61.2 | (48.5,73.9) |
| Rates reduce by 2% per year | 31.8 | (25.1,38.5) | 52.1 | (40.1,64.1) | 78.1 | (47.4,108.8) |

**References**

1 Royston P, Parmar MKB. Flexible parametric proportional-hazards and proportional-odds models for censored survival data, with application to prognostic modelling and estimation of treatment effects. *Stat Med* 2002; **21**: 2175–2197.

2 Jackson C. flexsurv : A Platform for Parametric Survival Modeling in R. *J Stat Soft* 2016; **70** (8): doi: 10.18637/jss.v070.i08.

3 Mandel M. Simulation-based confidence intervals for functions with complicated derivatives. *Am Stat* 2013; **67**: 76–81.

4 [Akaike H.](https://en.wikipedia.org/wiki/Hirotugu_Akaike) A new look at the statistical model identification. IEEE Transactions on Automatic Control, 1974; **19**: 716–723.
